# Supplementary material for: Face Coverings, Aerosol Dispersion and Mitigation of Virus Transmission Risk
Source: IEEE Open J Eng Med Biol. 2021 Jan 20;2:26–35. doi: 10.1109/OJEMB.2021.3053215 (PMC8545035; doi:10.1109/OJEMB.2021.3053215)
Supplement: A detailed description of the methodology, including (I) Instrumentation, (II) Data Processing, (III) Breathing Patterns, and (IV) Cough Generator. [file supp1-3053215.pdf]

# Supplementary Materials

## Face Coverings, Aerosol Dispersion and Mitigation of Virus Transmission Risk

**I. M. Viola*, B. Peterson, G. Pisetta, G. Pavar, H. Akhtar, F. Menoloascina, E. Mangano, K. E. Dunn, R. Gabl, A. Nila , E. Molinari, C. Cummins, G. Thompson, T. Y. M. Lo, F. C. Denison, P. Digard, O. Malik, M. J. G. Dunn, C. M. McDougall, F. V. Mehendale**

### I. Instrumentation

A high-speed CMOS camera (VEO 710L, Vision Research) with 1280 x 800 pixels$^2$ was used for recording. The camera operated in single-frame mode with a repetition rate of 100 Hz and exposure time of 9996.6 $\mu$s. Hence, each image is identified by a frame number. The camera was equipped with a 50 mm Nikon lens operating with f# = 11.

The camera imaged onto a patterned background placed at a distance $L$ = 2200 mm from the camera. The patterned background was printed onto a matte poster-board (1400 x 1000 mm$^2$). The background was generated using LaVision's Random Pattern Generator 1.3 software with dot size of 2.2 mm and a minimum dot distance of 1.1 mm. A blue LED-Flashlight 300 (LaVision) was used to illuminate the background. This LED unit is comprised of 72 high-power LEDs and operated with a pulse duration of 300 $\mu$s. A piece of sanded-down transparent acetate was placed in front of the LED unit to diffuse the illuminated light. A programmable timing unit (LaVision) was used to synchronize the LED light source with the high-speed camera. Acquisition and analysis of the BOS images were performed using commercial software (DaVis 10.1 by LaVision).

### II. Data Processing

The raw images were processed using the LaVision's DaVis 10 software. First, a time filter was applied to the raw images in a pre-processing stage to mitigate the effects of shot noise. The filter computes the intensity for each pixel from five consecutive images and assigns the maximum value to the pixel in the filtered image. The flow field was then visualised by evaluating the BOS displacement, which follows the motion of the dot pattern in the background on each image with respect to the reference image recorded before the test, where no flow was present (natural airflow in the room was minimised as far as possible). The software computes an average displacement value of the dots within a square window, called a subset, chosen in this case to be of 19 x 19 pixels$^2$. After calculating the displacement in the current subset, the process was repeated by shifting the subset by five pixels, until the entire image was scanned. The image displacement data was fitted with a first order polynomial which is then subtracted from the original BOS displacement map. This allows to eliminate the global bias in the BOS field which might be occurring due to motion and vibration or due to overall illumination variations. The contours of the resulting computed displacement are used to visualise the air flow.

### III. Breathing Patterns

Spirometry (Nuvoair Next) was performed on the person who performed all the tests shown in this paper, in line with ATS/ERS standards for spirometry [1]. The person is a co-author of this paper and he gave informed consent to undertake these measurements. These measurements were conducted by an Intensive Care Specialist using the Nuvoair Air Next, a CE certified and FDA cleared portable spirometer. Detailed results are available as Supplementary Material (Spirometry Results). Measurements of forced vital capacity were 1.29, 4.64, 1.65 litres over durations of 3.25, 2.09, 1.32 s for quite breathing, heavy breathing and coughing, respectively. The forced expired volumes in one second were 0.90, 4.33, 1.63 litres, respectively. The peak expiratory flows were 1.21, 8.99, 5.86 litres/second, respectively.

We also perform a reduced-order characterisation of the breathing patterns using BOS. At the beginning of a cough event, as air exits the subject's mouth, the schlieren signal is the strongest. Progression of the leading edge of the gas cloud can be clearly tracked within the first 1-3 frames when the schlieren signal is first observed near the subject's mouth. An estimate of the air velocity is calculated by recording the distance between the leading edge of the signal within successive frames and dividing by the time elapsed between the frames. This velocity estimation is inherently averaged across the line of sight of the camera. The velocities measured near the subject's mouth approximate the average peak velocity at the beginning of a cough. Conversely, for quiet and heavy breathing, we estimated the velocity as the maximum distance covered by the leading edge of the gas cloud within the field of view over the time it took to reach that point. This reduces the global bias in the BOS field, which may occur due to motion and vibration or due to overall illumination variations.

## IV. Cough Generator

The manikin enables repeatability of the tests when comparing different face coverings and it allowed increasing the temperature of the airflow to measure the displacement of the jet further downstream by moving the field of view.

The manikin's trachea is connected to a built-in-house breath/cough simulator. The system is designed to replicate the key aspects of human breath and cough by generating an air flow which is then released to the manikin's trachea at pre-defined time intervals. Air flow is generated from an air compressor for low velocity flow, i.e. simulation of quite breathing; while compressed air from a cylinder is used to simulate cough events. The dual air source allows to set the outlet air velocity in the whole range of velocities reported for human breath and cough. The system is connected to the mannikin's trachea via a remotely-controlled solenoid valve. In the case of breath simulation the valve is kept open for the entire duration of the experiment, while to simulate cough events the is controlled using a timed sequence which allows to open the valve for very short time intervals. This allows to simulate cough events 200 ms, in line with the literature [2].

The air entering the manikin's trachea is pre-heated using electrical heating elements to ensure an outlet flow from the manikin mouth of about 40°C. The air temperature within the room was 20-22°C. The plastic tube connecting the manikin to the breath simulator is insulated, to minimise the generation of noise during BOS measurements.

The manikin's mouth has fixed shape and an aperture of about 6.4 cm$^2$, while the real person mouth was open to about 3 cm$^2$ (note that the manikin and the person were used to perform different sets of tests and thus this difference is irrelevant). The manikin's nasal airway is sealed to prevent leakage.

## V. Front Throughflow and Main Leakage for Different Face Covers

**Table SM-I.** Quiet breath, human, sideways (95% CI < ±5°, ±2 cm).

| | | Front throughflow | | | | Brow jet | | | |
|---|---|---|---|---|---|---|---|---|---|
| **Mask** | **Test** | **Frame** | **Angle** (deg) | **Distance** (cm) | **H Dist.** (cm) | **Frame** | **Angle** (deg) | **Distance** (cm) | **V Dist** (cm) |
| none | 204 | 557 | 1 ±5 | >56 | >56 | not discernible | | | |
| surgical | 98 | 87 | 48 ±5 | 9±2 | 6±2 | 232 | 88±5 | >21±2 | >21±2 |
| FFP1 | 101 | 182 | 46±5 | 8±2 | 5±2 | not discernible | | | |
| FFP2 | 103 | 104 | -15±5 | 3±2 | 3±2 | not discernible | | | |
| handmade | 105 | not discernible | | | | not discernible | | | |
| respirator | 107 | 57 | -48±5 | 15±2 | 10±2 | not discernible | | | |
| heavy-duty commercial face shield | 109 | not discernible | | | | not discernible | | | |
| UoE lightweight 3DP face shield + opaque cover | 133 | not discernible | | | | not discernible | | | |

**Table SM-2.** Heavy breath, human, sideways (95% CI < ±5°, ±2 cm).

| | | Front throughflow | | | | Brow jet | | | |
|---|---|---|---|---|---|---|---|---|---|
| **Mask** | **Test** | **Frame** | **Angle** (deg) | **Distance** (cm) | **H Dist.** (cm) | **Frame** | **Angle** (deg) | **Distance** (cm) | **V Dist** (cm) |
| none | 198 | 181 | -7±5 | >55 | >55 | not discernible | | | |
| surgical | 99 | 80 | -24±5 | 28±2 | 25±2 | 67 | 58±5 | 18±2 | 15±2 |
| FFP1 | 102 | 197 | -18±5 | 20±2 | 19±2 | not discernible | | | |
| FFP2 | 104 | 95 | 12±5 | 20±2 | 19±2 | not discernible | | | |
| handmade | 106 | 41 | -11±5 | 14±2 | 14±2 | 32 | 32±5 | 20±2 | 11±2 |
| respirator | 36 | 135 | -54±5 | >27 | >16 | not discernible | | | |
| heavy-duty commercial face shield | 110 | 206 | -24±5 | 14±2 | 12±2 | not discernible | | | |
| UoE lightweight 3DP face shield + opaque cover | 134 | not discernible | | | | not discernible | | | |

**Table SM-III.** Coughing, manikin, sideways (95% CI < ±5°, ±2 cm).

| | | Front throughflow | | | | Crown jet | | | |
|---|---|---|---|---|---|---|---|---|---|
| **Mask** | **Test** | **Frame** | **Angle** (deg) | **Distance** (cm) | **H Dist.** (cm) | **Frame** | **Angle** (deg) | **Distance** (cm) | **V Dist** (cm) |
| none | 253, 254, 255 | 84, 72, 172 | -10±5 | 114±2 | 112±2 | not discernible | | | |
| surgical | 256 | 77 | -24±5 | 36±2 | 33±2 | 17 | 114±5 | >17 | >15 |
| FFP1 | 257 | 236 | 16±5 | 42±2 | 40±2 | 64 | 118±5 | >18 | >15 |
| FFP2 | 258 | 119 | -22±5 | 12±2 | 11±2 | 74 | 121±5 | >19 | >16 |
| handmade | 259 | 123 | 14±5 | 23±2 | 23±2 | 61 | 123±5 | >19 | >16 |
| respirator | 260 | 59 | -61±5 | >31 | >15 | 57 | 99±5 | >11 | >11 |
| heavy-duty commercial face shield | 261 | not discernible | | | | 70 | 82±5 | 13±2 | >6 |
| UoE lightweight 3DP face shield + opaque cover | 262 | not discernible | | | | 80 | 38±5 | 9±2 | >4 |

## Metadata

Metadata (>250 GB) is available on the Edinburgh DataShare at https://datashare.is.ed.ac.uk/handle/10283/3636. Data include, the spirometry tests for the human volunteer and, for each of the 244 tests undertaken, the measured raw data (camera pictures) and the processed data showing the displacements for each frame, and a video for ease of visualisation. In addition, for selected tests, including all of those for which quantitative data is provided in the paper, there are images with annotated measurements.

## References

[1] M. R. Miller, "Standardisation of spirometry," *Eur. Respir. J.*, vol. 26, no. 2, pp. 319–338, Aug. 2005.

[2] J. K. Gupta, C.-H. Lin, and Q. Chen, "Flow dynamics and characterization of a cough," *Indoor Air*, vol. 19, no. 6, pp. 517–525, Dec. 2009.
